# The Application of Pressure in Treatment of Uterine Diseases

**Published:** 1879-02

**Authors:** 


The Application of Pressure in Treatment of Uterine Diseases.—[In our September number we reprinted a paper by Prof. V. H. Taliaferro, of Atlanta, Ga., on the above subject. We subsequently tried the treatment as indicated, but the wool produced too much irritation, and treatment had to be suspended. We wrote to the Doctor, stating the case, and that portion of his reply relating to the tampon we take the liberty of presenting to our readers.—*Ohio Medical Record.*]

"I would advise when you have an irritable canal, and, indeed, in the beginning of treatment in all cases, to use cotton instead of wool. Wool is more irritating than cotton, and when you have a vagina at all irritable, cotton should be used. I have a patient now under treatment who always complains of wool, and in whom I substituted the cotton entirely. And even with the cotton, in these cases, care must be used and the effects watched until the vagina becomes thoroughly tolerant of the foreign substance. I sometimes suspend the treatment for two or three days, directing the patient to use hot water, with a little salt, as a vaginal douche twice or thrice daily during the time. This should be used while the patient is on her back, or, if "squatting," as they usually prefer, the labia should be grasped around the syringe nozzle and the vagina pumped full to distension, and then emptied—the process again and again repeated during the entire syringing. In this way only, in the sitting or stooping position, is the entire vagina with its vault douched with the water. After two or three days' rest to the vagina, and use of the water, the tampon is resumed, care being used to pack only the upper portion of the vagina at first, and then gradually inure the irritable canal to the foreign substance, and after awhile effectually destroy the irritable condition of the canal. A chronic irritability of the vaginal canal, and often a chronic inflammation, complicate the uterine trouble, and in such complications they must be removed, or at least greatly modified, before we can use the tampon effectively to the uterus. It sometimes occurs in healthy vaginæ, during the course of tamponing, that an occasional abrasion occurs. For these I never suspend treatment, but use a little cerate or iodoform ointment on a soft cloth and the cotton over it.

"In reference to the use of cotton in place of wool, I have become convinced, since the publication of my paper, that cotton is less irritating and a greater degree of pressure can be made with it. I am in the habit of using cotton to the upper two-thirds of the canal, and filling out with wool when I wish to make the greatest amount of pressure. The cotton should be prepared by boiling in soda water, and then washing thoroughly and carding into bats. The last water in which it is washed should contain a little carbolic acid. The first pledgets of cotton applied to the vaginal roof should contain, to saturation, glycerine of pure quality."

